# Deep Learning Derived Object Detection and Tracking Technology Based on Sensor Fusion of Millimeter-Wave Radar/Video and Its Application on Embedded Systems

**DOI:** 10.3390/s23052746

**Published:** 2023-03-02

**Authors:** Jia-Jheng Lin, Jiun-In Guo, Vinay Malligere Shivanna, Ssu-Yuan Chang

**Affiliations:** 1 Institute of Electronics, Nation Yang Ming Chiao Tung University, Hsinchu 30010, Taiwan; 2 Pervasive Artificial Intelligence Research (PAIR) Labs, National Yang Ming Chiao Tung University, Hsinchu 30010, Taiwan; 3 Wistron-NCTU Embedded Artificial Intelligence Research Center, National Yang Ming Chiao Tung University, Hsinchu 30010, Taiwan; 4 Department of Multimedia, Mediatek Inc., Hsinchu 30010, Taiwan

**Keywords:** millimeter-wave radar, depth sensor, sensor fusion, object detection and tracking, early fusion, deep learning

## Abstract

This paper proposes a deep learning-based mmWave radar and RGB camera sensor early fusion method for object detection and tracking and its embedded system realization for ADAS applications. The proposed system can be used not only in ADAS systems but also to be applied to smart Road Side Units (RSU) in transportation systems to monitor real-time traffic flow and warn road users of probable dangerous situations. As the signals of mmWave radar are less affected by bad weather and lighting such as cloudy, sunny, snowy, night-light, and rainy days, it can work efficiently in both normal and adverse conditions. Compared to using an RGB camera alone for object detection and tracking, the early fusion of the mmWave radar and RGB camera technology can make up for the poor performance of the RGB camera when it fails due to bad weather and/or lighting conditions. The proposed method combines the features of radar and RGB cameras and directly outputs the results from an end-to-end trained deep neural network. Additionally, the complexity of the overall system is also reduced such that the proposed method can be implemented on PCs as well as on embedded systems like NVIDIA Jetson Xavier at 17.39 fps.

## 1. Introduction

**Figure 1:**
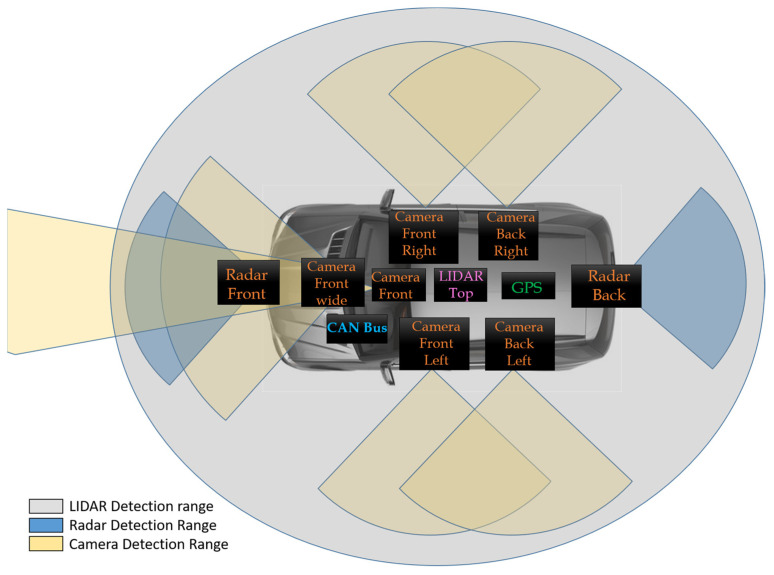
The various devices required in collecting the inputs in an ADAS system.

In recent years, the Advanced Driving Assistance System (ADAS) greatly promotes safe driving and might avoid dangerous driving events saving lives and damages to the infrastructure. Considering safety in autonomous system applications, it is crucial to accurately understand the surrounding environment under all circumstances and conditions. In general, autonomous systems need to estimate the positions and the velocities of probable obstacles and make decisions ensuring safety. The input data of the ADAS system is composed of various sensors, such as millimeter-wave (mmWave) radars, cameras, controller area networks (CAN) bus, light detection and ranging (LiDAR) and so on are utilized to help the road users perceive the surrounding environment and make correct decisions for safe driving. Figure 1 shows various equipment essential in an ADAS system. 

**Figure 2:**
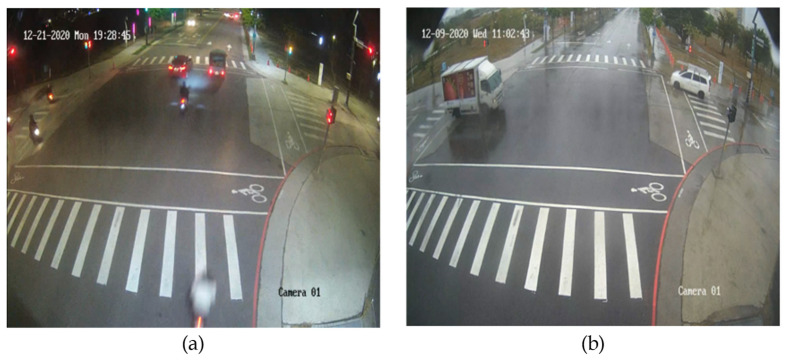
(**a**) Night scene; (**b**) Rainy day.

Vision sensors are the most common sensors around us and their applications are everywhere. They have many advantages, such as high resolution, high frame rate, and low hardware cost. As deep learning (DL) has become extremely popular [1,2], the importance of vision sensors has gradually peaked. Since the visual sensors can preserve the appearance information of the targets, they are best suited for DL technology. As aforementioned, it can be noted that camera-only object detection is widely utilized in a lot of fields for numerous applications, such as smart roadside units (RSU), self-driving vehicles, smart surveillance, etc. However, the results of object detection by the camera are severely affected by the ambient light and adverse weather conditions. Although the camera can distinguish the type of objects well, it cannot accurately obtain the physical characteristics such as the actual distance and velocity of the detected objects. In the ADAS industry, many companies with relevant research such as Tesla, Google, and Mobileye, use other sensors to design their self-driving cars to make up for the lack of camera failures in bad weather and lighting conditions such as nightlight, foggy, dusky, and rainy conditions as shown in Figure 2.

In contrast to the camera, the mmWave radars provide the actual distance and velocity of the detected object relative to the radar, and they can also provide the intensity of the object as a reference for identification, such that it convinces that the mmWave radar is a good choice to be employed together with camera for sensor fusion applications to yield better detection and tracking efficiency in all weather and lighting conditions. Compared to LiDAR, the mmWave radar has better penetration and is cheaper. Although mmWave radar has fewer point clouds than LiDAR, it is easy to use in the clustering algorithm to find the objects. When we can put the advantages of mmWave radar and camera to good use, these two sensors complement each other and provide a better perception capability compared to expensive 3-D LiDARs.

Basically, the three main fusion schemes have been proposed to use mmWave radar and camera together namely, (i) decision-level fusion, (ii) data-level fusion, and (iii) feature-level fusion, respectively [3]. For autonomous systems, different sensors can make up for the shortcomings of the others and overcome the worse situation through sensor fusion methods. Hence, we think that the radar and camera sensor fusion is better and more reliable for drivers than utilizing a single sensor like the radar-only sensor, or the camera-only sensor.

The following sections of the paper discuss the related works comprising of three existing mmWave radar and camera sensors fusion methods followed by the steps involved in the proposed early fusion technology of mmWave radar and camera sensors fusion, experimental results, and the conclusion.

### Motivation

This paper focuses on the early fusion of the mmWave radar and camera sensors for object detection and tracking as the late fusion of the mmWave radar and camera sensors belongs to decision-level fusion [4,5,6]. First, the mmWave radar sensor and camera sensor detect obstacles individually. Then, the prediction results from them are fused together to obtain the final output results. However, different kinds of detection noises are involved in the predictions of these two heterogeneous sensors. Therefore, how to fuse the prediction results of these two kinds of sensors is a great challenge encountered in the late fusion of the mmWave radar and camera sensors.

To solve the above problem, this paper proposes the early fusion of the mmWave radar and camera sensors which is also known as feature-level fusion. To begin with, we need to transform the radar point cloud from the radar coordinates to that of an image. In the process, we add information like the distance, velocity, and intensity of the detected objects from the radar points cloud to the radar image channels corresponding to different physical characteristics of the detected objects. Finally, we fuse the visual image and the radar image to a multi-channel array and utilize a DL object detection model to extract the information from both sensors. Through the object detection model, the early fusion on the mmWave radar and camera sensors learns the relationship between the data from the mmWave radar and camera sensors, which can not only solve the problem of decision-level fusion but also solve the problem of detection in harsh environments when using the camera only.

This paper is organized as follows. Section 2 reviews the related works, which include the three existing mmWave radar and camera sensor fusion methods, related deep learning object detection models, some radar signal processing algorithms, and adopted image processing methods followed by the introduction of the proposed early fusion technology on the mmWave radar and camera sensors in detail in Section 3. Section 4 depicts our experiments and results along with the conclusion and future works in Section 5.

## 2. Related Work

### 2.1. Types of Sensor Fusion

**Figure 3:**
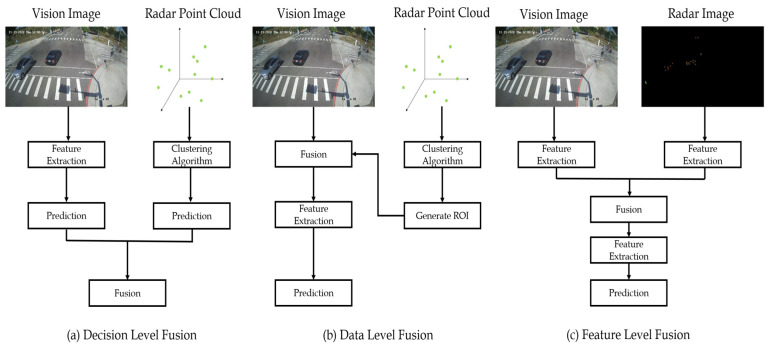
Three types of sensor fusion on the mmWave radar and camera sensors: (**a**) decision-level fusion, (**b**) data-level fusion, and (**c**) feature-level fusion.

As discussed in the previous section, the methods of sensor fusion [7,8] are broadly categorized into three types viz, (a) decision-level fusion, (b) data-level fusion, and (c) feature-level fusion as shown in the respective flowcharts in Figure 3.

For the decision-level fusion [9], there are two heterogeneous types of prediction results from mmWave radar and camera that are fused to obtain the final results. Considering that the data types from the mmWave and camera sensors are heterogeneous, there are no good methods to fuse their respective prediction results, which are involved in their detection noises.

The second sensor fusion method is a data-level fusion [10,11,12], in which we first need to cluster the radar point cloud. Then, find the positions of the clustering points to generate the regions of interest (ROIs) where there may be objects to be detected. Finally, through the ROIs, we need to extract the corresponding image patches from the input image and utilize objection detection models to obtain the final predicted results. This fusion method requires a lot of valid radar points, so some objects cannot be detected if there are no valid radar points on them. Although the data-level fusion method can reduce the operational complexity and solve the decision problem in decision-level fusion, it is not suitable for the autonomous system from safety considerations.

The final sensor fusion method is a feature-level fusion [13,14,15]. Usually, in the feature-level fusion method, the radar point cloud is transformed from the radar coordinates to the image coordinates, namely the radar image, as shown in Figure 3. Then, the radar image and the corresponding vision image are fused and extracted based on the features of the DL models. The feature-level fusion can not only solve the decision-making problem in the decision-level fusion but also learns the relationship between the mmWave radar and vision image using the DL models.

The contributions of the early sensor fusion method proposed in this paper are: (i) It employs the fusion of the mmWave radar and the RGB camera sensor for more precise object detection and tracking compared to either camera-only or sensor-only methods. (ii) It can be used in an ADAS system for object detection and tracking as well as be applied to a smart Road Side Unit (RSU) in smart transportation to monitor real-time traffic flow for warning dangerous situations for all road users.

### 2.2. YOLO v3 Model

For YOLO v3 [1], it has some good characteristics like bounding box prediction, no softmax, feature pyramid networks (FPN), etc. The authors have used logistic regression to predict the confidence score of each object in the bounding box. The purpose is to distinguish the targets and the background. The IOU value of the bounding boxes and the ground truth are used as the criterion to evaluate the detection efficiency. One of the important features of YOLO v3 is that it does not use the softmax to classify each box, because the softmax imposes an assumption that each box contains only one category whereas, in practice, different objects possess overlapping labels. For example, it is predicted that boys belong to the category of people. For the autonomous system field, there are many multi-label scenarios, so the softmax is not suitable for multi-label classification. In addition, YOLO v3 makes predictions on three different scales, namely 13 × 13, 26 × 26, and 52 × 52, in which there are three bounding boxes predicted on each scale. This approach helps YOLO v3 to better detect small objects, and the up-sampling technology helps the network learn subtle features for detecting small objects. As autonomous systems require real-time and accurate judgment, the YOLO v3 model becomes an ideal choice.

### 2.3. YOLO v4 Model

YOLO v4 [2] is an improvement of YOLO v3, which improves the input terminal during the training phase so that training can yield good results on a single GPU. For instance, the mosaic used in YOLO v4 refers to the CutMix data augmentation method [16] proposed in 2019, but CutMix only uses two images for stitching. While mosaic data augmentation [17] uses four images to achieve random scaling, random cropping, and random arrangement for stitching. In the normal training processes, the average precision of small targets is generally much lower than that of medium and large targets. The COCO dataset [18] also contains a large number of small targets, but the crucial challenge is that the distribution of small targets is not uniform. Therefore, mosaic data augmentation can balance the proportion of small, medium, and large targets. Thus, the backbone of YOLO v4 uses Cross Stage Partial Network (CSPNet) [19] reduces repetitive gradient learning greatly enhancing the learning ability of the network. Although the model architecture of YOLO v4 is more complicated than that of YOLO v3, YOLO v4 uses a lot of 1 × 1 convolutions to reduce the number of calculations and increase the processing speed. Therefore, the YOLO v4 model is also suitable for the autonomous field.

### 2.4. Clustering

Based on our proposed system, the radar that we use is the Frequency Modulated Continuous Wave (FMCW) radar [20]. The FMCW radar emits continuous waves with varying frequencies during the frequency sweep period. The echo reflected by the object has a certain frequency difference from the transmitted signal. The distance information between the target and the radar can be obtained by measuring the frequency difference. The frequency of the difference frequency signal is relatively low, so the hardware processing is relatively simple. Therefore, the FMCW radar is suitable for data acquisition and digital signal processing.

K-means clustering [21] is the most common and well-known clustering method. K-means clustering is similar to the concept of finding the center of gravity. First, it divides the radar point cloud into k groups, and randomly selects k points to be the center of the cluster. Second, it classifies each point to its nearest cluster center. Third, it recalculates the cluster centers of each group. Finally, steps two and three are repeated until a stable k cluster is found. However, the problem of K-means clustering is that we cannot know the number of clusters and the number of repetitions prior. The data distribution and the initial location of the cluster centers affect the number of repetitions. Therefore, for autonomous applications, we think that K-means clustering is not the most suitable clustering algorithm.

**Figure 4:**
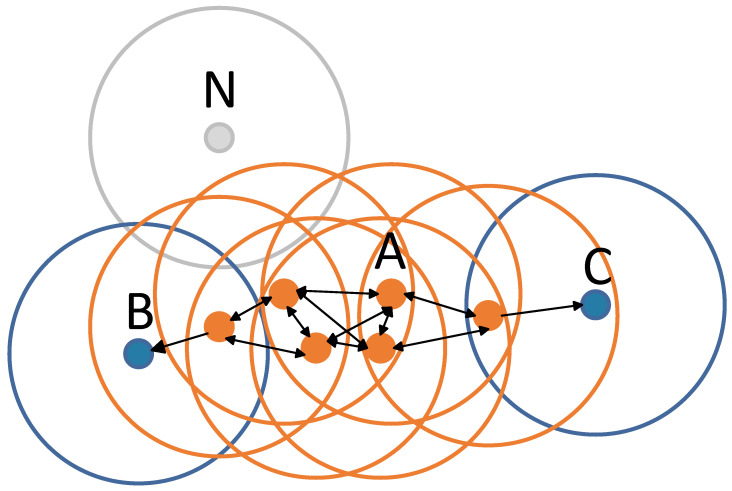
Schematic diagram of DBSCAN.

Density-based spatial clustering of applications with noise (DBSCAN) [22] clustering algorithm is one of the most commonly used clustering analysis algorithms. In DBSCAN, there are two main parameters, distance (ε) and the minimum number of points (minPts), as shown in Figure 4. In step 1, it first decides the parameters and determines the ε and minPts. In step 2, it selects a random sample as the center point and draws a circle with the ε set from step 1. If the number of samples in the circle is greater than minPts, this sample is the core point and the marker can reach any point in the circle. If the number of samples in the circle is less than minPts, then this sample is a non-core point and cannot reach any point. In step 3, we repeat step 2 for each sample until all samples are over the center point. In step 4, we divide the connected sample points into a group, and other outlier points can be divided into different groups by examining whether they can be reached individually.

Compared to K-means clustering, DBSCAN does not require a pre-declared number of clusters. It is possible to find clusters of any shape and even find a cluster that encloses but does not connect to another cluster. DBSCAN can also distinguish noise with only two parameters and is almost insensitive to the order of the points in the database. Therefore, for applications in the autonomous domain, we believe that DBSCAN is more suitable than the K-means for clustering in the proposed method.

## 3. The Proposed Method

### 3.1. Overview

**Figure 5:**
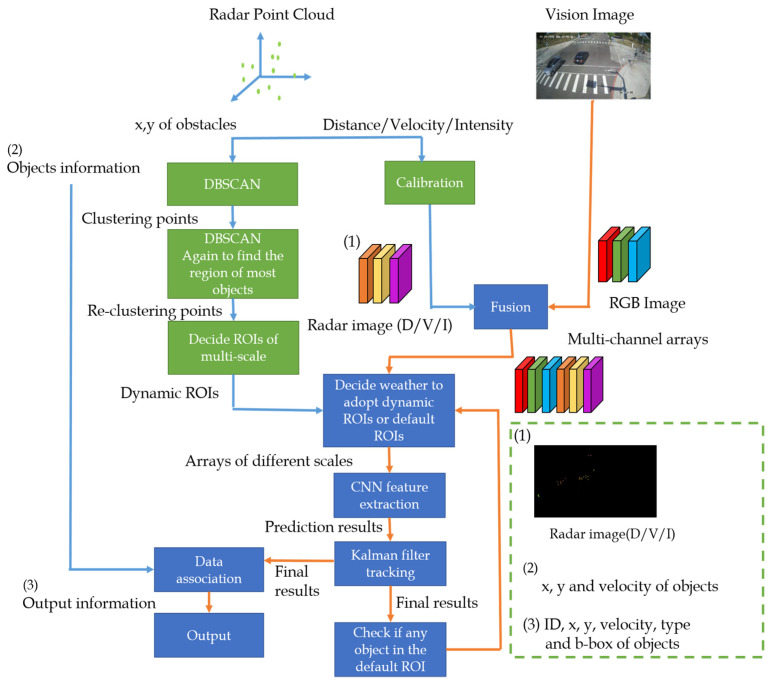
The overall architecture of the proposed method.

Figure 5 depicts the overall architecture of the proposed early sensor fusion method. The x and y positions and velocity indicate the relative 2-D distance (x, y) and velocity between the proposed system and the detected object. First, we will get the mmWave radar point cloud and the corresponding image. Then, the radar point cloud will be clustered and the radar and camera calibration is performed. The purpose of clustering is to find the areas where objects are really present and to filter out the noise of radar. The RGB image is represented as three channels, R, G, and B, while the radar image is represented as D, V, and I. All six channels are concatenated into a multi-channel array in the early fusion process. In Section 3.2, the clustering process and the related parameter adjustment are described in detail. The obtained clustering points are then clustered again to find out where most of the objects are present so that we can determine the ROIs of our multi-scale object detection. In Section 3.3, the radar and camera calibration is implemented to get the radar image that corresponds to the input image. In Section 3.4, Section 3.5 and Section 3.6, a detailed description of how to perform early fusion on the radar and camera sensors and how to determine our ROIs for multi-scale object detection are given, respectively. Then, in Section 3.7, we will explain how the Kalman filter is used for object tracking.

### 3.2. Radar Clustering

**Figure 6:**
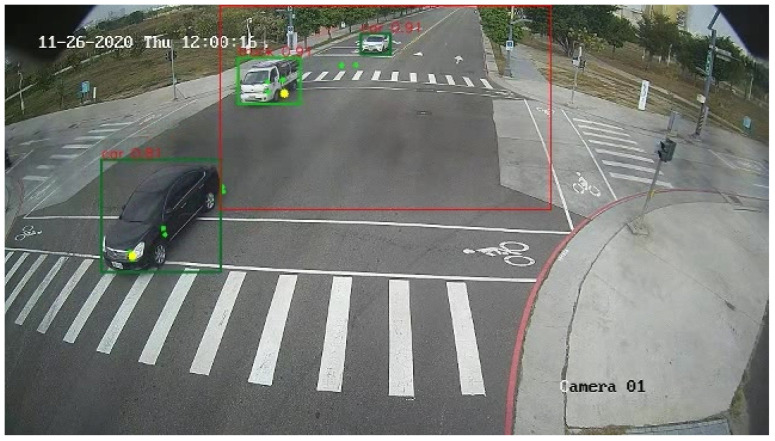
The results of using DBSCAN: green dot: mmWave radar point cloud; yellow dot: clustering points.

We have to set two parameters first before using DBSCAN. One is the minimum point that forms the range of each clustering point. The other one is the minimum distance to form each cluster point range. We have experimentally set 4 as the minimum point and 40 cm as the minimum distance. Figure 6 shows the effect after using DBSCAN. In Figure 6, the green dot is the mmWave radar point cloud and the yellow dot is the clustering point after DBSCAN. The red rectangular box is the ROI of multi-scale object detection, which will be introduced in detail in the later section.

Furthermore, we need to find out the area where most of the objects appear in each frame. Therefore, DBSCAN is performed again after radar and camera calibration for the above clustering points. Since the number of points in the cluster is fewer, we have experimentally set 1 as the minimum point and 400 pixels as the minimum distance.

### 3.3. Radar and Camera Calibration

**Figure 7:**
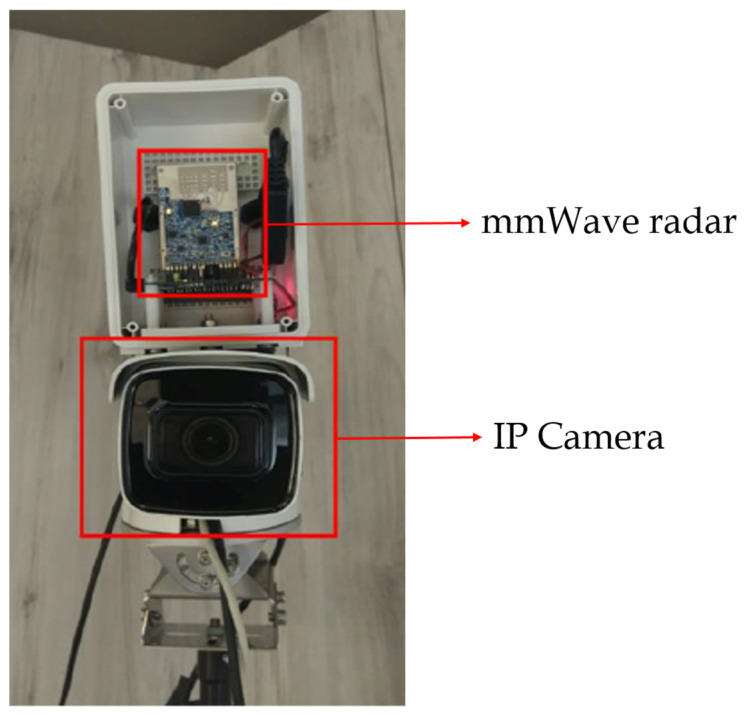
The setting of the early fusion on the mmWave radar and camera sensors.

To make the proposed system easier to set up and calculate the angle faster, we have derived the camera/radar calibration formula based on [23]. Figure 7 shows the early fusion device of mmWave and camera sensors. The following is a detailed description of the entire radar and camera calibration process.

**Figure 8:**
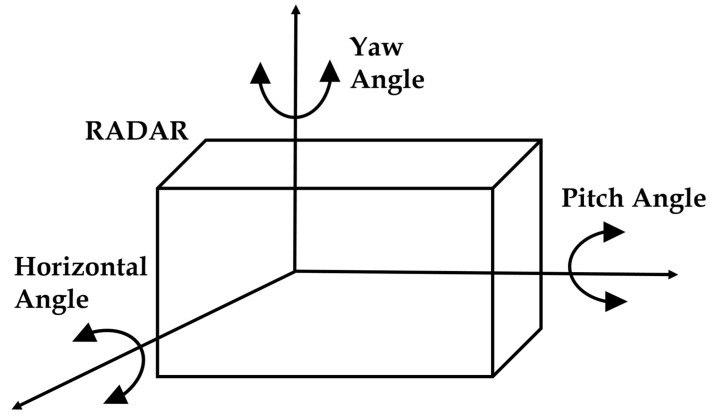
Schematic diagram of mmWave radar installation.

**Figure 9:**
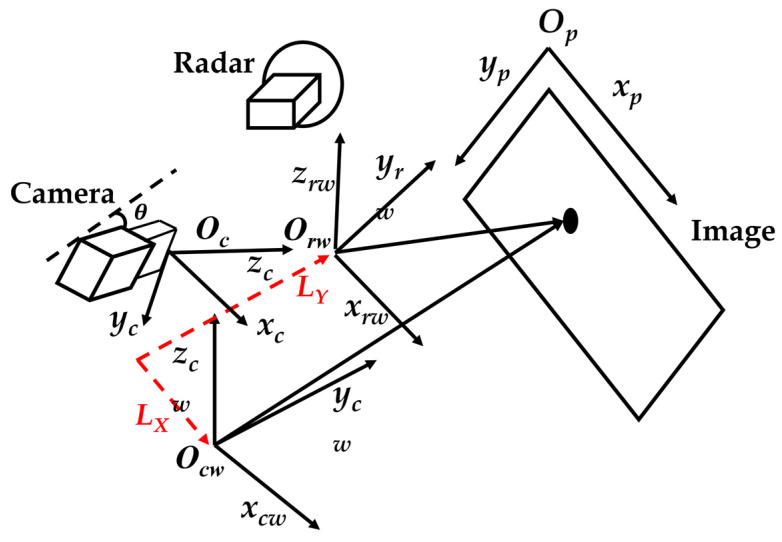
The relationship of mmWave radar, camera, and image coordinates.

For the calibration, we have three angles to be calculated, namely yaw angle, horizontal angle, and pitch angle as shown in the schematics of Figure 8. For the convenience of installation and more convenient to calculate the other angles, we have set the horizontal angle to zero. Figure 9 shows the relationship between mmWave radar, camera, and image coordinates. First, we need to transform the radar world (r coordinate O_rw_-x_rw_y_rw_z_rw_ to the camera world coordinate O_cw_-x_cw_y_cw_z_cw_. Then, the camera world coordinate O_cw_-x_cw_y_cw_z_cw_ is transformed into the camera coordinate O_c_-x_c_y_c_z_c_. Finally, we transform the camera coordinate O_c_-x_c_y_c_z_c_ to the image coordinate O_p_-x_p_y_p_.

**Figure 10:**
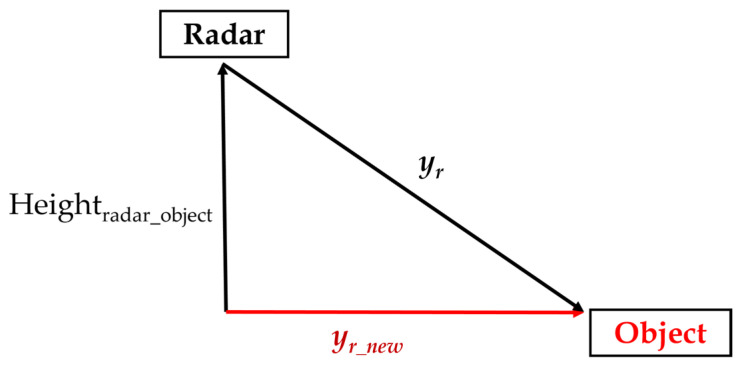
The height relation of mmWave radar and object.

First, we must transform the radar coordinate O_r_-x_r_y_r_ to radar world coordinate O_rw_-x_rw_y_rw_z_rw_. Since the radar only has 2-D coordinates and no *z*-axis information, we can only get the relevant 2-D distance (x, y) between the radar and the object. Therefore, to get the radar world coordinates, we need to calculate the radar yaw angle and the height difference between the radar and the object. In the case of the height difference, since the radar does not have *z*-axis information, we need to consider the height difference between the radar and the object to calculate the projected depth distance “y_r_new_” correctly. In Figure 10, we show the height relationship of mmWave radar and the object. The parameter “y_r_” is the depth distance from mmWave radar and the “Height_radar_object_” is the height difference between the mmWave radar and the object. The function shows how we obtain the projected depth distance “y_r_new_” using Equation (1).

(1) $$y_{r\_\mathrm{new}}=\sqrt{y_{r}^{2}-{\mathrm{Height}}_{\mathrm{radar}\_\mathrm{object}}^{2}}$$

**Figure 11:**
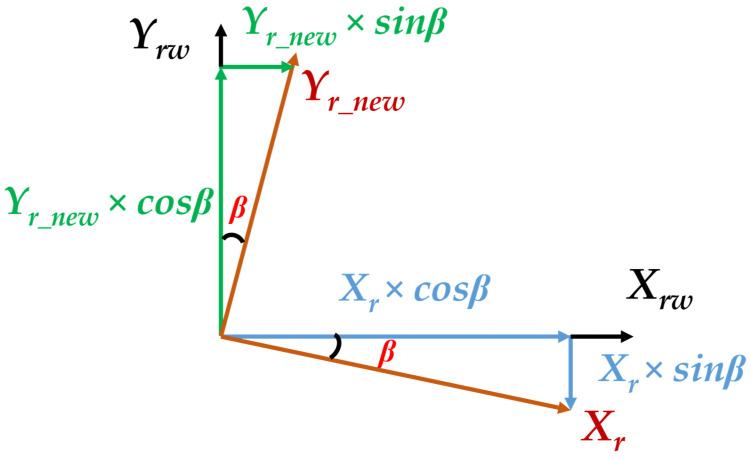
The relationship of radar coordinate, radar world coordinate, and yaw angle.

When we get the projected depth distance “y_r_new_”, we also need to go through the yaw angle “β” to transform from the radar coordinate O_r_-x_r_y_r_ to the radar world coordinate O_rw_-x_rw_y_rw_z_rw_. Figure 11 and Equation (2) show the relationship between radar coordinate, radar world coordinate, and yaw angle.

(2) $$\begin{array}{c} x_{\mathrm{rw}}=x_{r}\times \cos\beta +y_{r\_\mathrm{new}}\times \sin\beta \\ y_{\mathrm{rw}}=\left(-x_{r}\times \sin\beta \right)+y_{r\_\mathrm{new}}\times \cos\beta \end{array}$$

The above steps help us to convert from the radar coordinate system to the radar world coordinate system. Then we need to transform from the radar world coordinate system to the camera coordinate system O_cw_-x_cw_y_cw_z_cw_, as shown in Equation (3). In Equation (3), “L_x_” and “L_y_” are the horizontal and vertical distances between the mmWave radar sensor and camera sensor, respectively. Thus, “L_x_” and “L_y_” are preset to zero.

(3) $$\begin{array}{c} x_{\mathrm{cw}}=x_{\mathrm{rw}}-L_{x}\\ y_{\mathrm{cw}}=y_{\mathrm{rw}}+L_{y} \end{array}$$

After transferring to the camera world coordinate O_cw_-x_cw_y_cw_z_cw_, we need to transform the camera world coordinate to the camera coordinate O_c_-x_c_y_c_z_c_. The function shown in Equation (4) is used for transferring the camera world coordinate to the camera coordinate. The parameters “H” and “θ” are the height and pitch angle of the camera sensor, respectively.

(4) $$\left[\begin{array}{c} x_{c}\\ y_{c}\\ z_{c} \end{array}\right]=\left[\begin{array}{ccc} 1 & 0 & 0\\ 0 & -\sin\theta & -\cos\theta \\ 0 & \cos\theta & \sin\theta \end{array}\right]\left[\begin{array}{c} x_{\mathrm{cw}}\\ y_{\mathrm{cw}}\\ z_{\mathrm{cw}} \end{array}\right]+\left[\begin{array}{c} 0\\ H\cos\theta \\ H\sin\theta \end{array}\right]$$

**Figure 12:**
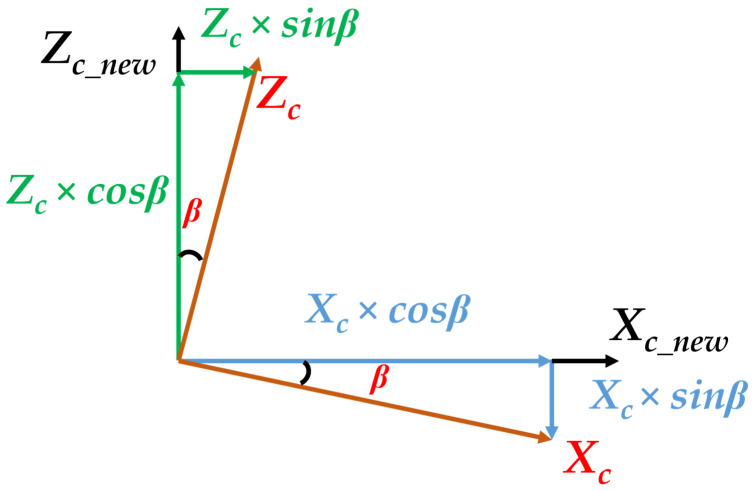
The relationship of camera coordinate, new camera coordinate, and yaw angle.

Then, similar to the above conversion of radar coordinate to radar world coordinate, we also regard the yaw angle “β” effect of the camera coordinate. Figure 12 shows the relationship between the camera coordinate, the new camera coordinate, and the yaw angle. Thus, the function shows the equation to transfer the original camera coordinate to the new camera coordinate influenced by “β” as in Equation (5).

(5) $$\left\{\begin{array}{c} x_{c\_new}=x_{c}\times \cos\beta +z_{c}\times sin\beta \\ y_{c\_new}=y_{c}\\ z_{c\_new}=(-x_{c}\times \sin\beta )+z_{c}\times cos\beta \end{array}\right.$$

Finally, we can get Equation (6) by the new camera coordinate. The function helps us to transfer the new camera coordinate to the image coordinate as in Equation (6). The parameters “f_x_” and “f_y_” are the focal length, and the “c_x_” and “c_y_” are the principal points of the camera sensor. We can then calculate the four parameters using the MATLAB camera calibration toolbox.

(6) $$\left\{\begin{array}{c} x_{p}=\frac{x_{c\_\mathrm{new}}}{z_{c\_\mathrm{new}}}\times f_{x}+c_{x}\\ y_{p}=\frac{y_{c\_\mathrm{new}}}{z_{c\_\mathrm{new}}}\times f_{y}+c_{y} \end{array}\right.$$

**Figure 13:**
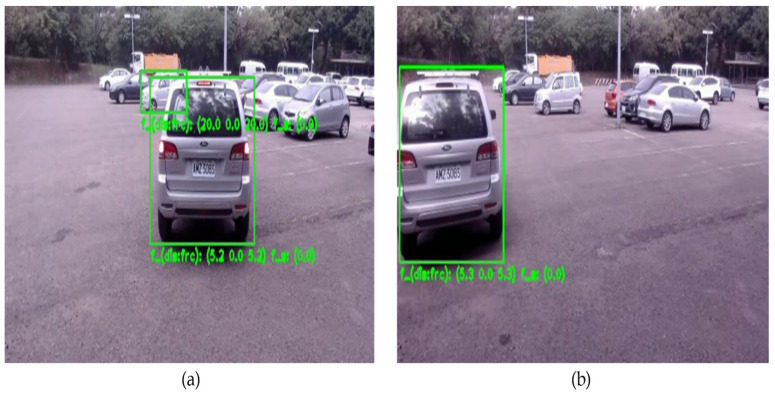
(**a**) Car drives directly in front of our system; (**b**) Car drives 25 degrees to the left of our system.

Figure 13 shows the experiments conducted to measure the accuracy of the radar and camera calibration. First, we measure the latitude and longitude of the system with a GPS meter and then use the center point position behind the vehicle as the ground truth measurement point. These two points are used to obtain the ground truth distance by using the haversine formula [24]. To estimate the radar distance, we take the radar point cloud information and calibrate it with the camera, and then use the clustering and data association algorithm [25] to find out which radar points belong to the vehicle. Finally, the radar points belonging to the vehicle are averaged to obtain the radar estimated distance. Table 1 shows the results of the radar and camera calibration, which indicates that the distance error of the calibration is at most 2% ranging from 5 m to 45 m. 

### 3.4. Radar and Camera Data Fusion

As mmWave radar and camera sensors are heterogeneous and are used by the deep learning models for object detection, it is essential to transform the radar point cloud information to the image coordinate system for the early fusion of the radar and camera sensors using the radar and camera calibration method discussed in Section 3.3. In this way, we can not only make the models learn the sizes and shapes of the objects but also let them learn the physical characteristics of the objects resulting in better detection results.

**Figure 14:**
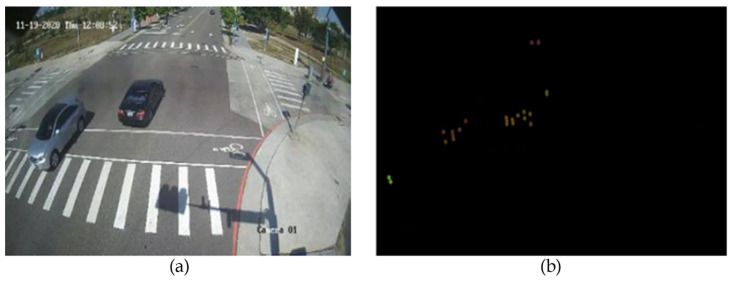
(**a**) Camera image; (**b**) radar image.

In our experiments, we use the distance “D”, velocity “V” and intensity “I” of the mmWave radar as individual channels, and the DVI pairs are arranged and combined with the camera images. Since the pixel values of the image range from 0 to 255, we need to experimentally set the maximum value of DVI. We want to make the difference in physical characteristics bigger, so we have a conversion equation for DVI design as shown in Equation (7) where the parameter “d” means the distance of mmWave radar, and the maximum value is set to 90 m. The parameter “v” is the velocity of mmWave radar and the maximum value is set to 33.3 m/s. As there is no negative pixel value, we use the absolute value of the velocity in Equation (7). As for the parameter “I” is considered, TI IWR6843 mmWave radar only provides the signal-to-noise ratio (SNR) and noise, hence we need to convert them into the intensity “I” whose maximum value is set to 100 dBw. Figure 14 shows the RGB image from the vision sensor and the radar image from the mmWave radar sensor. For the radar image, if the pixel values exceed the value 255, we consider them to be equal to 255. In addition, we set all pixels where there are no radar points equal to zero. When we have the camera image and the radar image, we combine the two images to get multi-channel arrays.

(7) $$\left\{\begin{array}{c} D=d*2.83\\ V=\left|v\right|*7.65\\ I=(10\log_{10}(10^{\mathrm{SNR}*0.01}*(P_{\mathrm{Noise}}*0.1)))*2.55 \end{array}\right.$$

### 3.5. Dynamic ROI for Multi-Scale Object Detection

**Figure 15:**
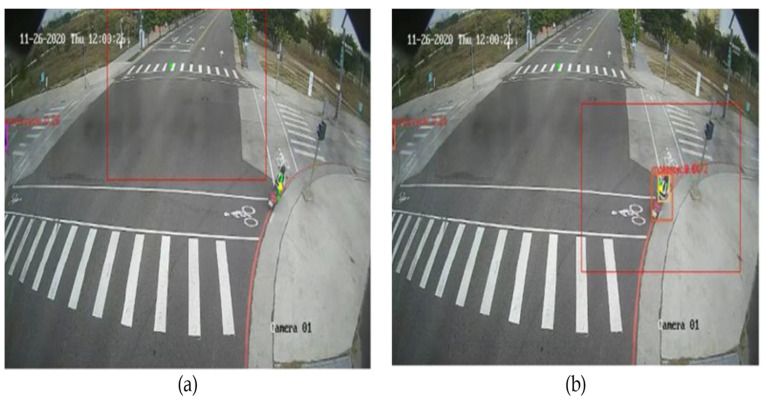
(**a**) Fixed ROI; (**b**) Dynamic ROI.

This section proposes the method of employing mmWave radar to dynamically find ROI and apply it to multi-scale object detection. Thus, we also compare the difference between fixed ROI and dynamic ROI as shown in Figure 15.

We found that in the original multi-scale object detection method, we could only set the default ROI at the beginning because we could not know the position of the objects explicitly in advance. Therefore, we propose to use the mmWave radar sensor to find the area with the most objects and set it as the new ROI. As discussed in Section 3.2, we use the clustering algorithm to cluster the radar point cloud and find the presence of objects. Then, we cluster the clustering points again to find out which area has the most objects. This region is set as the new ROI that we have to find using the mmWave radar point cloud. Figure 15 shows the advantages of dynamic ROI. When there is no object in the default ROI, the dynamic ROI we proposed can find the area where objects may appear followed by the successful detection of objects.

### 3.6. Object Detection Model

For the ADAS applications, the object detection models must be capable of operating in real-time and detect various objects ranging from small objects at a distance to near, bigger objects. Therefore, we selected the YOLOv3 and YOLOv4 as our desired object detection convolutional neural network (CNN) models. As the inputs must be the fusion of mmWave radar sensors and camera sensors and the available open datasets are comprised of only image data, we recorded our own dataset including both radar data and image data. Additionally, we need to label the dataset thus collected by ourselves, the available open datasets are unsuitable for training the proposed model. 

**Figure 16:**
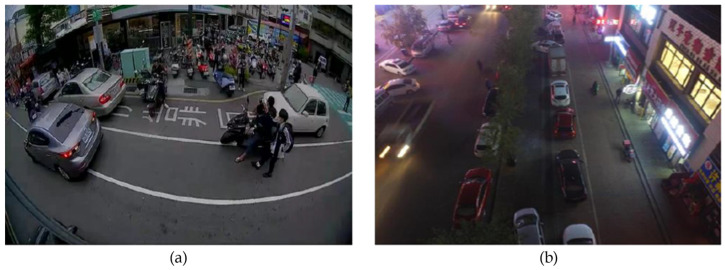
The examples of datasets we adopted: (**a**) Bling spot, and (**b**) VisDrone.

To solve this problem, we used camera-only datasets, such as the COCO dataset [16] and the VisDrone dataset [26] to increase the amount of training data. The Chinese characters in Figure 16a is the traffic rule craved on the road and in Figure 16b is the name of a business unit. Since these open datasets are only camera data, we set all pixel values in the radar channels to zero. Figure 16 shows examples of the datasets. Considering our applications also require RSU perspectives, we used VisDrone and the blind-spot datasets to fit our real-life traffic scenario requirements.

### 3.7. Tracking

Using the object detection model, we obtain the bounding box and detect the type of object, such as a person, car, motorcycle, or truck. We select the bounding boxes as the input of the trackers. Unlike the late fusion on the radar and camera sensors, we do not do tracking of radar data and the bounding boxes of the camera individually. We only need to track the bounding boxes generated by the object detection model [27,28].

**Figure 17:**
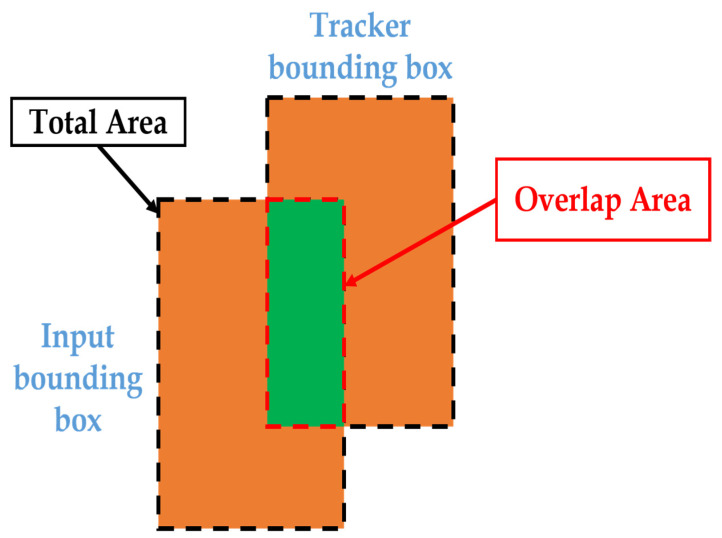
The schematic diagram of IoU.

However, we still need to carry out certain pre-processing steps before feeding the bounding boxes to the trackers. The function given in Equation (8) shows the definition of the intersection of union (IoU) which is the overlapped area divided by the total area. Figure 17 shows the schematic diagram of IoU. The IoU input includes the bounding boxes of the tracker and object detection model. When the IoU value is higher than the set threshold, we can treat both as the same object. Based on the RSU application field characteristics, we adopt the Kalman filter to implement the tracking ensuring the trackers keep their motion information to solve the ID switch issue.

(8) $$\left\{IoU=\frac{overlap\;area}{total\;area}\right.$$

## 4. Experimental Evaluation

### 4.1. Sensor Fusion Equipment

**Figure 18:**
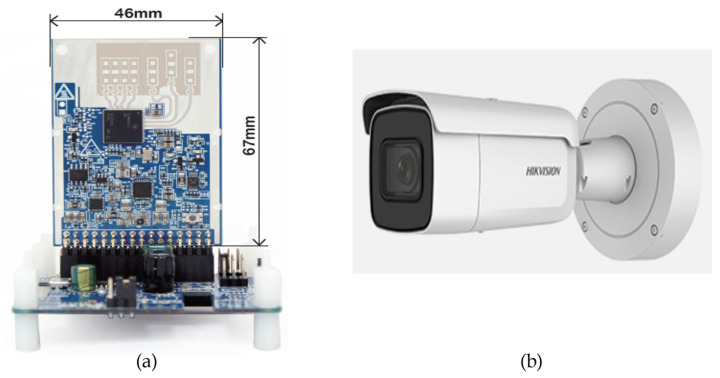
(**a**) TI IWR6843 mmWave radar [29]; (**b**) IP-2CD2625F-1ZS IP camera.

In the proposed work, the TI IWR6843 is chosen as the mmWave radar. The IWR6843 mmWave radar has four receive antennas (RX) and three transmit antennas (TX). As this radar sensor has its own DSP core to process the radar signal, we can directly obtain the radar point cloud for experiments. Figure 18a shows the TI IWR6843 mmWave radar sensor employed in this paper. Since our application is set up at a certain height above the vehicle overlooking the ground, we choose this radar sensor with a large vertical field of view of 44° that facilitates this application.

The IP-2CD2625F-1ZS IP camera shown in Figure 18b is employed in the proposed work. It offers 30 fps with a high image resolution of 1920 × 1080. The waterproof, dustproof, and clear imaging against strong backlight characteristics of the camera aids to overcome the impact of the harsh environment in the ADAS scenarios.

**Figure 19:**
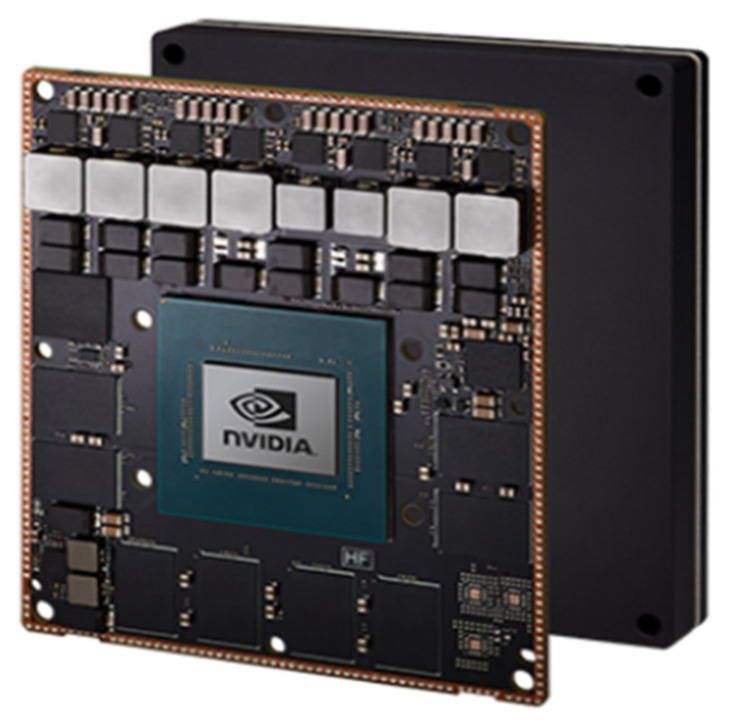
NVIDIA Jetson AGX Xavier [30].

We choose NVIDIA Jetson AGX Xavier [30] as the embedded platform to demonstrate the portability of the proposed early fusion system on the radar and camera sensors. NVIDIA Jetson AGX Xavier comes with a pre-installed Linux environment. With the NVIDIA Jetson AGX Xavier, as shown in Figure 19, we can easily create and deploy end-to-end deep learning applications. We can think of it as an AI computer for autonomous machines, offering the GPU workstation in an embedded module under 30 W. Therefore, NVIDIA Jetson AGX Xavier enables our proposed algorithm to be conveniently implemented for low-power applications.

### 4.2. Implementation Details

**Figure 20:**
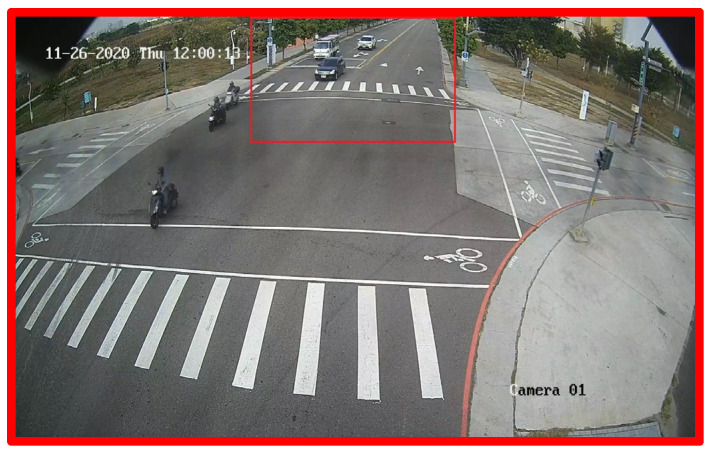
The ROI for multi-scale object detection.

**Figure 21:**
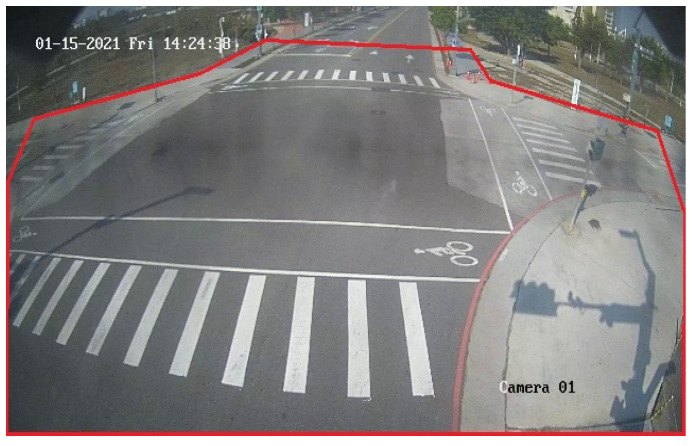
The ROI of calculation accuracy.

We have collected 8285 frames of training data as radar/camera datasets by using a multi-threading approach to capture the latest radar and camera data in each loop and used 78,720 frames of camera-only datasets to make up for the lack of data. For testing purposes, we have collected 896 images for each of the four conditions namely, morning, noon, evening, and night. Figure 20 shows the ROI for the multi-scale object detection which has a big ROI covering the entire image, and the small ROI is used for the distant region. As the mmWave radar used in this work only detects around 50 m in a given field, we set the accuracy measurement in the 50-m range, as shown in Figure 21.

The accuracies of YOLOv3 and YOLOv4 models with and without the camera-only datasets and the comparison of the effects with and without multi-scale object detection are tabulated in Section 4.3. The input sizes are set to be 416 × 416 × N, where “N” is the channels of input arrays. The confidence thresholds are set at 0.2 for pedestrians, 0.2 for bicycles, 0.2 for motorcycles, 0.4 for cars, and 0.4 for full-size vehicles. The IoU threshold is set to 0.5.

### 4.3. Evaluation on YOLOv3

Table 2 shows the accuracy of the YOLOv3 model with camera-only datasets and multi-scale object detection. 

### 4.4. Evaluation on YOLOv4

Table 3 shows the accuracies of the proposed method on the YOLOv4 model with camera-only datasets and multi-scale object detection. 

### 4.5. Comparison between YOLOv3 and YOLOv4

Table 4 shows a comparison of the best fusion of radar and camera between the YOLOv3 and YOLOv4 models. The left-hand side represents the training data of the models without the camera-only data, and the right-hand side represents the training data of the models with the camera-only data. From Table 4, we can know that the YOLOv3 model yields the best results when the input type is RGB + DV and multi-scale object detection is used.

### 4.6. Proposed System Performance

Table 5 shows the accuracy comparison of the FP32, the FP16 RGB + DV models, the proposed system in INT8, and the late fusion method [4]. We can see that the proposed system has the best recall because of the addition of the Kalman filter. But the precision is reduced because of the ghost frame. 

**Figure 22:**
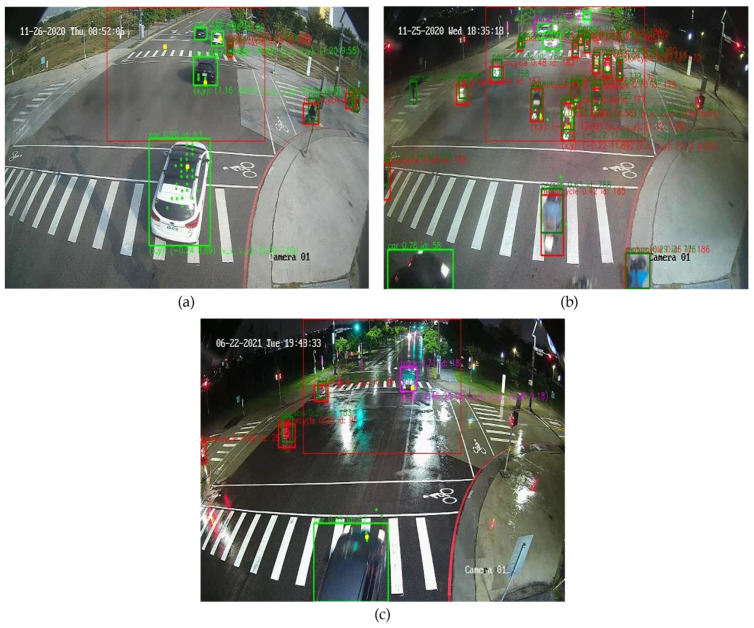
The demo of the proposed system: (**a**) morning, (**b**) night, and (**c**) rainy night.

Compared to the late fusion method, the proposed system is better in the aspects of precision, recall, and mAP. In addition, the average operational performance of the proposed system is 17.39 fps which is better than the average operational performance of the late fusion method which has 12.45 fps when implemented on the NVIDIA Jetson AGX Xavier. Table 6 shows the comparison of the proposed system and the late fusion method in rainy conditions. With the early fusion of DV and RGB data from the mmWave sensor and RGB sensor. It shows that the proposed system is significantly improved in overall mAP by 10.4% relative to the late fusion method. Figure 22 shows the demonstration of the result images for various scenarios that the proposed system can offer in terms of id, type, x-y coordinate, and velocity of the detected objects.

## 5. Conclusions

The proposed mmWave radar/camera sensor early fusion algorithm in this paper is mainly designed to solve the decision-making challenges encountered in late sensor fusion methods and the proposed method improves the detection and tracking of objects while attaining real-time operational performance. The proposed system combines the advantages of mmWave radar and vision sensors. Compared with the camera-only object detection model, Table 2 and Table 3 show a significant improvement in the detection accuracies of the proposed design.

Compared to the radar/camera sensor late fusion method, the proposed system not only has better overall accuracy but also has a faster operating performance of about 5 fps. Unlike the camera-only object detection model, the proposed system offers additional relative x-y coordinates and the relative velocity of the detected objects. For the RSU applications, the proposed system can provide accurate relative positions of objects. Table 1 shows the distance errors of the proposed system, which is, at most, a 2% error rate between the ranges of 5 m to 45 m.

However, there is scope to carry out future work to improve the proposed early sensor fusion method. That is, the mmWave radar proposed in this paper outputs around 30 to 70 radar points. In complex scenes, this amount of radar points may not be enough. To overcome this challenge, we can improve the radar equipment in future work to obtain more radar information about the position and velocity information of the detected objects which is the future work of the proposed method.

## Figures and Tables

**Table 1:** The distance error of radar and camera calibration from 5 m to 45 m.

	Angle (Deg)	Ground Truth (m)	Radar Estimation (m)	Radar (Error)
Point 1	0	5.10	5.20	1.96%
Point 2		9.58	9.5	−0.84%
Point 3		13.92	13.90	−0.14%
Point 4	25	5.00	4.90	−2.00%
Point 5		19.26	18.90	−1.87%
Point 6		34.04	33.90	−0.41%
Point 7		45.42	46.00	1.28%

**Table 2:** Evaluation on YOLOv3 with camera-only datasets and multi-scale object detection where the readings in red highlight the highest value in each row.

	Class	RGB	RGB + D	RGB + V	RGB + I	RGB + DV	RGB + DI	RGB + VI	RGB + DVI
Precision (%)	All	68.7	71.2	69.4	67.4	77.5	71.1	65.7	74.4
	Person	42.0	44.0	35.1	39.6	50.6	39.5	46.3	44.7
	Bicycle	56.3	65.2	62.5	62.0	76.7	65.0	62.9	64.7
	Car	95.0	95.2	95.0	94.7	96.2	95.2	94.8	95.2
	Motorcycle	74.5	78.5	76.3	78.6	78.6	72.3	74.0	79.0
	F-S vehicle	75.6	73.0	78.1	62.0	86.5	83.6	50.7	88.4
Recall (%)	All	59.4	59.4	57.5	60.7	61.5	59.3	60.1	61.1
	Person	52.0	50.0	40.0	50.4	51.0	49.6	53.9	47.9
	Bicycle	9.0	11.8	7.5	12.0	12.1	10.9	10.4	8.0
	Car	93.4	93.7	93.1	91.9	94.9	94.1	89.8	94.6
	Motorcycle	66.2	69.8	69.2	69.9	68.6	65.9	66.7	75.3
	F-S vehicle	76.5	71.8	77.7	79.5	81.2	75.7	79.5	79.5
mAP (%)	All	49.8	51.5	50.2	51.3	54.3	50.3	50.4	52.6
	Person	26.5	31.0	20.8	50.2	51.3	54.3	50.3	52.6
	Bicycle	7.7	10.0	6.7	10.6	11.4	8.9	8.7	7.3
	Car	91.4	91.0	91.7	89.3	92.8	92.9	87.3	92.9
	Motorcycle	51.4	57.3	59.0	57.7	56.8	50.8	51.7	61.9
	F-S vehicle	72.0	68.2	72.7	72.0	78.1	70.6	72.5	73.8

**Table 3:** Evaluation on YOLOv4 with camera-only datasets and multi-scale object detection where the values in red are the highest value in each row.

	Class	RGB	RGB + D	RGB + V	RGB + I	RGB + DV	RGB + DI	RGB + VI	RGB + DVI
Precision (%)	All	72.0	73.8	68.6	69.2	68.6	65.3	63.8	64.0
	Person	44.3	37.3	39.6	36.9	37.1	41.7	44.1	36.3
	Bicycle	76.9	78.3	67.5	73.4	68.5	72.3	64.2	64.4
	Car	95.8	95.0	95.7	95.9	96.0	96.0	95.0	95.5
	Motorcycle	78.9	72.4	75.2	73.8	70.3	74.0	76.7	76.1
	F-S vehicle	64.1	86.1	65.0	66.0	71.3	42.8	38.8	47.4
Recall (%)	All	57.4	56.0	58.8	57.7	57.2	58.8	57.3	56.9
	Person	42.2	43.9	50.5	46.0	47.2	49.9	45.2	41.2
	Bicycle	8.4	10.3	8.1	9.1	8.8	7.5	8.2	6.8
	Car	91.0	94.3	91.9	91.9	92.2	85.8	85.2	87.5
	Motorcycle	63.9	54.7	60.9	58.6	54.6	66.4	65.8	64.2
	F-S vehicle	81.4	77.0	82.7	83.2	83.2	84.2	82.7	84.7
mAP (%)	All	48.9	47.5	48.8	50.2	47.9	46.6	44.9	45.0
	Person	24.9	20.8	25.7	23.6	22.9	25.1	24.7	21.3
	Bicycle	7.8	9.9	7.2	8.5	7.5	6.7	7.2	5.0
	Car	90.2	92.7	90.0	89.8	90.8	84.3	83.8	86.2
	Motorcycle	52.8	42.1	49.4	50.4	44.0	53.3	55.2	53.4
	F-S vehicle	69.0	71.9	71.6	78.5	74.1	63.8	53.6	59.4

**Table 4:** Evaluation between YOLOv3 and YOLOv4 with highest values highlighted in red.

	Class	RGB + VI (YOLOv3, 1ROI)	RGB + DVI (YOLOv3, 2ROI)	RGB + V (YOLOv4, 1ROI)	RGB VI (YOLOv4, 2ROI)	RGB + DVI (YOLOv3, 1ROI)	RGB + DV (YOLOv3, 2ROI)	RGB + I (YOLOv4, 1ROI)	RGB + I (YOLOv4, 2ROI)
Precision (%)	All	79.5	61.7	82.9	62.1	77.2	77.5	81.1	69.2
	Person	56.2	36.8	59.8	36.7	56.5	50.6	55.0	36.9
	Bicycle	72.0	73.5	93.8	55.0	81.1	75.7	73.4	73.4
	Car	94.5	81.9	94.2	86.3	92.8	96.2	95.7	95.9
	Motorcycle	85.6	68.7	81.6	74.5	84.0	78.6	84.1	73.8
	F-S vehicle	88.9	47.3	85.1	57.8	71.6	86.5	97.1	66.0
Recall (%)	All	54.4	57.9	47.8	53.5	49.8	61.5	46.6	57.7
	Person	40.0	40.2	33.5	36.2	37.6	51.0	32.5	46.0
	Bicycle	4.0	6.6	2.7	1.3	4.4	12.1	5.4	9.1
	Car	92.9	90.0	89.8	87.9	93.0	94.9	93.4	91.9
	Motorcycle	63.7	75.1	49.4	65.2	67.7	68.6	51.4	58.6
	F-S vehicle	71.3	77.2	63.6	77.0	46.3	81.2	50.5	83.2
mAP (%)	All	49.2	44.4	42.7	44.5	43.6	54.3	42.8	50.2
	Person	26.5	20.4	21.0	19.2	22.9	32.4	20.3	23.6
	Bicycle	3.9	5.9	2.5	1.1	4.4	11.4	5.4	8.5
	Car	89.7	86.1	86.0	83.4	90.5	92.8	91.0	89.8
	Motorcycle	56.2	61.2	43.4	55.8	57.2	56.8	46.9	50.4
	F-S vehicle	70.0	48.4	60.8	63.2	43.0	78.1	50.2	78.5

**Table 5:** The comparison of the FP32, FP16, the proposed system, and the late fusion method where the highest values in each row is highlighted in red.

	Class	RGB + DV (FP32) (YOLOv3, 2ROI)	RGB + DV (FP16) (YOLOv3, 2ROI)	RGB + DV (INT8) (YOLOv3, 2ROI)	RGB + Radar (Late Fusion) (YOLOv3, 2ROI)
Precision (%)	All	77.5	77.5	73.1	48.2
	Person	50.6	50.7	44.8	37.5
	Bicycle	75.7	74.3	66.4	15.1
	Car	96.2	96.3	95.5	94.2
	Motorcycle	78.6	78.7	75.7	65.6
	F-S vehicle	86.5	87.2	83.3	28.5
Recall (%)	All	61.5	61.5	62.2	61.8
	Person	51.0	50.6	50.6	52.7
	Bicycle	12.1	11.9	12.7	6.5
	Car	94.9	94.8	95.5	92.9
	Motorcycle	68.6	68.8	68.7	71.3
	F-S vehicle	81.2	81.2	83.7	85.6
mAP (%)	All	54.3	54.2	54.1	47.5
	Person	32.4	32.1	29.6	28.1
	Bicycle	11.4	11.0	11.2	28.1
	Car	92.8	92.8	93.5	90.6
	Motorcycle	56.8	56.9	57.0	56.8
	F-S vehicle	78.1	78.1	79.1	57.3

**Table 6:** The comparison of the proposed system and late fusion method on rainy days in which the values in red indicate the highest value in each row.

	**Class**	**RGB + DV (Proposed)** **(YOLOv3, 2ROI)**	**RGB + Radar (Late Fusion)** **(YOLOv3, 2ROI)**
Precision (%)	All	86.4	92.5
	Person	77.2	87.2
	Bicycle	n/a	n/a
	Car	98.2	96.7
	Motorcycle	79.3	91.7
	F-S vehicle	91.0	94.6
Recall (%)	All	87.0	76.5
	Person	80.0	51.4
	Bicycle	n/a	n/a
	Car	96.9	92.5
	Motorcycle	81.4	75.5
	F-S vehicle	89.6	86.6
mAP (%)	All	84.2	73.8
	Person	71.4	47.8
	Bicycle	n/a	n/a
	Car	95.7	91.0
	Motorcycle	79.0	71.9
	F-S vehicle	86.9	84.5

n/a = not measurable.

## Data Availability

The publicly available data set can be found at: https://cocodataset.org/#home (accessed on: 5 January 2021), and https://github.com/VisDrone/VisDrone-Dataset (accessed on: 5 January 2021).
